# Eyelid Complications in Subciliary Versus Transconjunctival Approaches to Orbital and Zygomaticofacial Fractures: A Meta-Analysis

**DOI:** 10.3390/jcm14186431

**Published:** 2025-09-12

**Authors:** Yu-Yen Chen, Yun-Ju Lai, Tai-Yuan Chen, Yung-Feng Yen

**Affiliations:** 1 Department of Ophthalmology, Taichung Veterans General Hospital, Taichung 407219, Taiwan; yuyenchen.phd@gmail.com; 2 Doctoral Program in Translational Medicine, National Chung Hsing University, Taichung 402202, Taiwan; 3 School of Medicine, National Yang Ming Chiao Tung University, Taipei 112304, Taiwan; lailai841081@yahoo.com.tw; 4 Program of Biomedical Informatics and Data Sciences, School of Medicine, Johns Hopkins University, Baltimore, MD 21218, USA; 5 School of Medicine, Chung Shan Medical University, Taichung 402306, Taiwan; 6 Division of Endocrinology and Metabolism, Department of Internal Medicine, Puli Branch of Taichung Veterans General Hospital, Nantou 545402, Taiwan; 7 Department of Health Care Management, National Taipei University of Nursing and Health Sciences, Taipei 112303, Taiwan; 8 School of Medicine, College of Medicine, Taipei Medical University, Taipei 110301, Taiwan; zxc49852@gmail.com; 9 Section of Infectious Diseases, Taipei City Hospital, Heping Fuyou Branch, Taipei 100058, Taiwan; 10 Institute of Public Health, National Yang Ming Chiao Tung University, Taipei 112304, Taiwan

**Keywords:** orbital fracture, zygomatic fracture, facial bone fracture, subciliary, transconjunctival, eyelid complications

## Abstract

**Background/Objectives**: Eyelid complications such as ectropion, entropion, scleral show, and visible scarring can lead to significant cosmetic and functional concerns following orbital and zygomaticofacial fracture repair. The subciliary and transconjunctival approaches are commonly used, but their relative complication risks remain debated. This study aimed to compare the risk of postoperative eyelid complications between these two approaches. **Methods**: A systematic review and meta-analysis were conducted in accordance with the PRISMA guidelines. The study protocol was registered with PROSPERO (CRD420251089460). PubMed, EMBASE, and the Cochrane Library were searched for studies published between 1 January 1990 and 15 June 2025. Data on study design, patient demographics, and complication rates were extracted following the PRISMA guidelines. The Newcastle–Ottawa scale was used for quality assessment. A random effects meta-analysis was performed using Comprehensive Meta-Analysis software (v4.0), and pooled odds ratios (ORs) with 95% confidence intervals (CIs) were calculated. **Results**: Nineteen studies involving 2103 patients (1062 subciliary; 1041 transconjunctival) were included for analysis. The subciliary approach was associated with significantly higher risks of ectropion (OR = 2.94; 95% CI: 1.63–5.31), scleral show (OR = 2.33; 95% CI: 1.12–4.84), and visible scar (OR = 5.62; 95% CI: 1.65–19.18). In contrast, the transconjunctival approach carried a higher risk of entropion (OR = 0.17; 95% CI: 0.07–0.42). Between-study heterogeneity and publication bias were minimal. **Conclusions**: As compared with the transconjunctival approach, the subciliary incision is associated with higher risks of ectropion, scleral show, and scarring, whereas the transconjunctival approach carries a greater risk of entropion. These findings may help guide surgical decision-making and patient counseling regarding postoperative outcomes.

## 1. Introduction

Orbital and zygomaticofacial fractures are common in high-energy trauma, such as motor vehicle accidents, physical assault, and industrial incidents [1]. Surgical intervention is frequently required to restore the anatomical integrity, orbital function, and cosmetic appearance of these fractures. The infraorbital rim, orbital floor, and zygomatic bone are typically accessed via external or internal surgical approaches, among which the subciliary and transconjunctival techniques are the most widely used [2].

Although both the subciliary and transconjunctival approaches have long been used to manage orbital or zygomatic fractures, there is an ongoing debate regarding which technique is associated with superior surgical outcomes [3,4]. The subciliary approach, introduced in the mid-20th century, provides excellent exposure but might be associated with a risk of postoperative ectropion, visible scarring, and scleral show [5]. In contrast, the transconjunctival approach has gained popularity due to its hidden incision, which minimizes visible scarring. However, concerns remain regarding entropion, limited exposure, and technical complexity, particularly in the context of extensive zygomaticomaxillary complex fractures [2,6].

Postoperative eyelid complications can result not only in cosmetic concerns but also in an increased risk of ocular surface diseases, such as conjunctivitis or corneal ulcers, which require medical or surgical intervention [7,8]. Previous studies have compared the risk of postoperative eyelid complications between the subciliary and transconjunctival approaches [3,6,9,10,11,12,13,14]. However, most of these studies included fewer than 50 patients per group, which makes it difficult to determine accurately the incidence or relative risk of eyelid complications. Although published review articles or meta-analyses have suggested that the transconjunctival approach is associated with a lower risk of ectropion and visible scarring but a potentially higher risk of entropion, several studies published in the past two years were not included in those analyses [5,15,16,17].

Given the clinical importance of minimizing postoperative eyelid deformities, we conducted this meta-analysis to systematically compare the incidence of eyelid complications. We evaluated specific complications of interest including ectropion, entropion, scleral show, and visible scars. This study integrates the latest available evidence to help surgeons better understand the risks associated with each surgical approach. This information can support more informed, evidence-based discussions with patients during preoperative planning for maxillofacial and oculoplastic surgery.

## 2. Materials and Methods

### 2.1. Search Strategy

We conducted this study according to the Preferred Reporting Items for Systematic Reviews and Meta-Analyses (PRISMA) guidelines provided in PRISMA 2020 checklists. The study was registered in PROSPERO on 7 July 2025 and the protocol including amendments can be accessed in the PROSPERO database (CRD420251089460). The ethical review board approval or participant informed consent was waived. We searched PubMed, EMBASE, and Cochrane databases for studies published from 1 January 1990 to 15 June 2025, using the following keywords: (zygomatic OR zygomaticomaxillary OR facial) AND fracture AND ectropion. We screened studies by first examining the titles and abstracts and then scrutinizing the full texts. We also manually searched the relevant literature in the bibliographies.

### 2.2. Inclusion and Exclusion Criteria

We included only peer-reviewed journal articles. The inclusion criteria were original prospective or retrospective clinical studies that compared eyelid complications after subciliary and transconjunctival approaches for orbital or zygomaticofacial fractures. We excluded reviews, meta-analyses, or studies with fewer than ten cases in either group. Two researchers (Y.-Y. Chen and T.-Y. Chen) independently assessed the articles. A third researcher (Y.-J. Lai) was consulted if consensus was not reached.

### 2.3. Quality Assessment

We applied the Newcastle–Ottawa scale as the evaluation tool for methodological quality, which included cohort adequacy selection, study comparability, and outcome assessment. Two researchers (Y.-Y. Chen and T.-Y. Chen) independently evaluated the quality of the included articles. When discrepancies occurred, a third researcher (Y.-J. Lai) reassessed the data and made the final decision.

### 2.4. Data Extraction

The following data were tracked from each included article: first author, year of publication, number/age of participants, and duration of follow-up. We also recorded the percentage of postoperative complications, including ectropion, entropion, scleral show, and scar.

### 2.5. Statistical Analysis

We performed the meta-analysis using the Comprehensive Meta-Analysis software, version 4.0 (Biostat, Englewood, NJ, USA). First, we calculated the odds ratios (ORs) for each complication (ectropion, entropion, scleral show, and scar) between the subciliary and transconjunctival groups in each study. Then, we pooled the ORs from each study to obtain the overall ORs. Thus, we could then identify the surgery that was favored. We determined the heterogeneity among the studies using the I^2^ statistic, with an I^2^ statistic of ≥50% representing high heterogeneity. Funnel plots and Egger’s test were used to assess publication bias. We also conducted a sensitivity analysis to assess whether the exclusion of any single study would alter the statistical significance of our findings.

## 3. Results

### 3.1. Search Results

**Figure 1:**
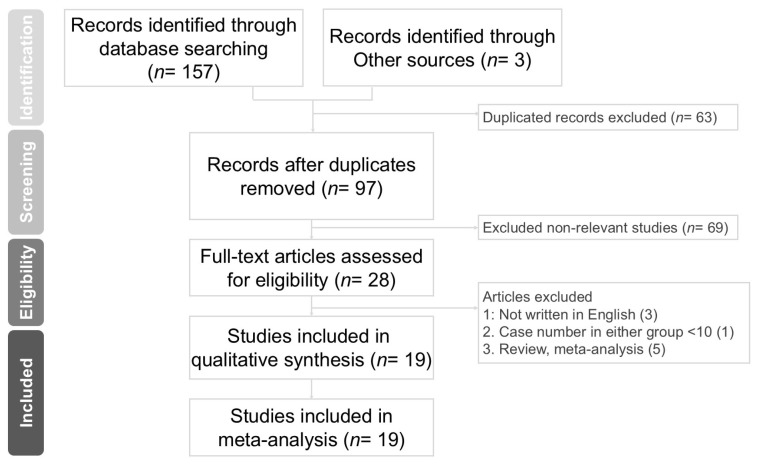
Preferred reporting items for systemic reviews and meta-analyses (PRISMA) flow diagram for searching and identifying included studies.

Figure 1 shows the PRISMA flow diagram. The PRISMA 2020 checklists is presented in Supplementary Table S1. We initially identified 160 studies. After eliminating duplicated articles (*n* = 63), we removed nonrelevant studies by screening titles and abstracts (*n* = 69). We then performed a full-text review. Review articles (*n* = 2), meta-analysis (*n* = 3), and studies with fewer than 10 cases in each group (*n* = 1) were excluded. The excluded studies and reasons are presented in Supplementary Table S2. We also excluded papers that were not written in English (*n* = 3). Finally, we included 19 studies in our meta-analysis [3,9,10,11,12,13,16,18,19,20,21,22,23,24,25,26,27,28,29].

### 3.2. Evaluation of the Quality of Included Studies

As shown in Table 1, quality assessment using the Newcastle–Ottawa scale indicated that all included studies had high methodological quality, with six studies achieving the maximum score of nine stars and the remaining receiving eight stars. The most critical item was comparability evaluation, which is a weak point of retrospective studies without randomization. The high score achieved by all included studies ensures the robustness and reliability of our meta-analysis.

### 3.3. Characteristics of the Included Studies

Table 2 summarizes the characteristics of the included studies. Of the included studies, 13 were retrospective, 5 were randomized, and 1 was a prospective nonrandomized clinical study. A total of 2103 patients were enrolled, with 1062 patients in the subciliary group and 1041 in the transconjunctival group.

### 3.4. Outcome Assessment

**Figure 2:**
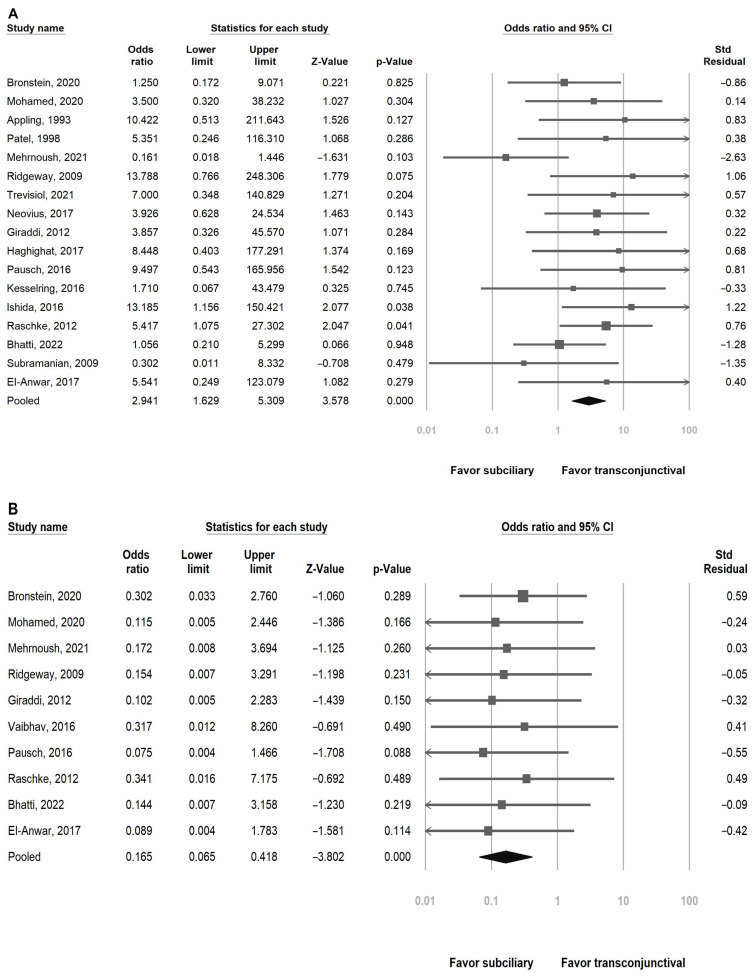

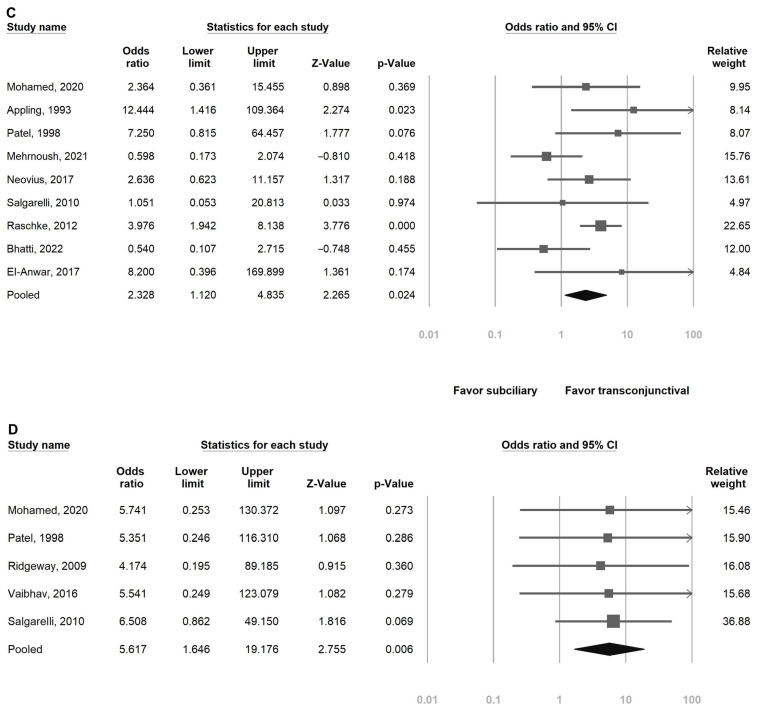
Forest plots comparing the risk of eyelid complications between the subciliary and transconjunctival approaches: (**A**) ectropion, (**B**) entropion, (**C**) scleral show, and (**D**) scar formation [3,9,10,11,12,13,16,18,19,20,21,22,23,24,25,26,27,28,29].

Table 3 presents the complications according to the subciliary and transconjunctival approaches in each study. The main complications were ectropion, entropion, scleral show, and scar. The majority of the included studies (15 of 19) involved orbital floor reconstruction [3,9,10,11,12,13,16,19,20,21,22,23,24,25,26]. Figure 2 shows the pooled analyses comparing the risk of these complications between the subciliary and transconjunctival approaches. All *p* values were less than 0.05.

#### 3.4.1. Ectropion

Seventeen studies reported postoperative ectropion [3,9,10,11,12,13,16,20,21,22,23,24,25,26,27,28,29]. There was a significant increase in the pooled risk of ectropion in patients who underwent the subciliary approach compared with the transconjunctival approach (OR = 2.94, 95% confidence interval [CI]: 1.63–5.31).

#### 3.4.2. Entropion

We included ten studies in the analysis of postoperative entropion [9,10,12,16,18,20,21,26,27,29]. The pooled results showed a significantly lower risk of entropion in patients who underwent the subciliary approach compared with those who received the transconjunctival approach (OR = 0.17, 95% CI: 0.07–0.42).

#### 3.4.3. Scleral Show

Data were available in nine studies to assess scleral show [3,10,11,12,19,20,22,27,29]. The meta-analysis revealed a marginally significant increase in the risk of postoperative scleral show with the subciliary approach compared with the transconjunctival approach (OR = 2.33, 95% CI: 1.12–4.84).

#### 3.4.4. Scar

Standardized evaluation scales commonly used for assessing lower eyelid scars include the Visual Analog Scale (VAS), the Patient and Observer Scar Assessment Scale (POSAS), the Vancouver Scar Scale (VSS), and the Manchester Scar Scale (MSS). Table 4 summarizes the scar assessment methods applied in the included studies. Among them, only one study utilized an internationally standardized scale (VAS) [28]. One study relied on a patient-reported satisfaction scale [18], which is subjective and presents outcomes as either satisfactory or unsatisfactory. The studies by Patel, Ridgway, and Salgarelli employed a binary visible scar assessment, reporting solely the presence or absence of a scar [3,16,19]. Mohamed’s study adopted the simplified Feldman scar esthetic score, which is a non-standardized, single-dimension tool that evaluates scar visibility only [10].

A total of five studies contributed to the analysis of scar, and the subciliary approach was associated with a significantly higher risk of postoperative scar compared with the transconjunctival approach (OR = 5.62, 95% CI: 1.65–19.18) [3,10,16,18,19].

### 3.5. Heterogeneity and Publication Bias

All analyses revealed low between-study heterogeneity, with I^2^ values of 3.54%, <0.001%, 43.19%, and <0.001%, corresponding to ectropion, entropion, scleral show, and scar, respectively. All *p* values were greater than 0.05.

**Figure 3:**
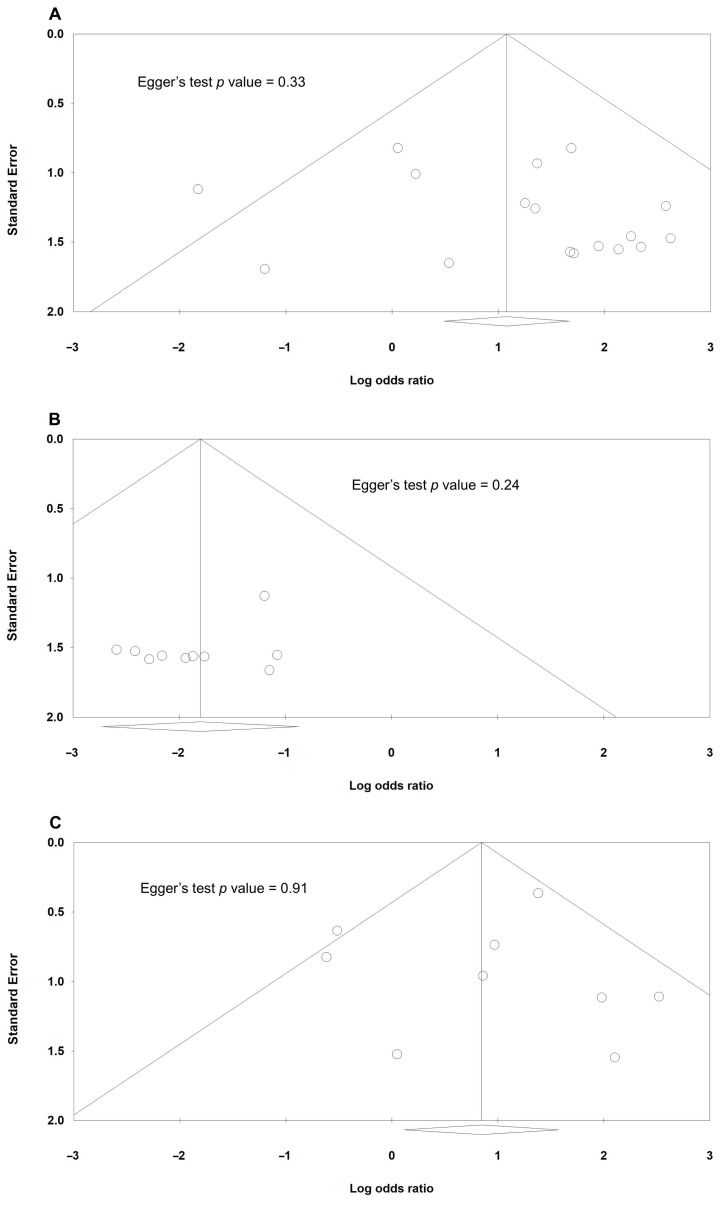

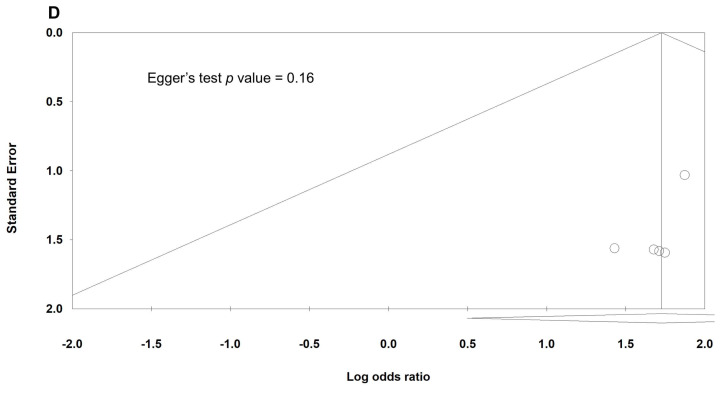
Funnel plots demonstrating no significant publication bias based on Egger’s test for: (**A**) ectropion (*p* value = 0.33), (**B**) entropion (*p* value = 0.24), (**C**) scleral show (*p* value = 0.91), and (**D**) scar (*p* value = 0.16).

With regard to publication bias, Figure 3 shows the funnel plots of all analyses. The *p* values of Egger’s test were 0.33, 0.24, 0.91, and 0.16, corresponding to ectropion, entropion, scleral show, and scar, respectively; therefore, there were no significant publication biases.

### 3.6. Sensitivity Analysis

The results showed that removing any single study did not affect the significance of our findings.

## 4. Discussion

In this meta-analysis, we included 19 studies focusing on the comparison of eyelid complications between the subciliary and transconjunctival approaches for orbital and zygomaticofacial fractures. The group undergoing the subciliary approach had a significantly higher risk of postoperative ectropion, scleral show, and scar as compared with the transconjunctival group. Conversely, patients who received the transconjunctival approach had a significantly higher risk of entropion than those who underwent the subciliary approach did.

With regard to the forest plot of ectropion, 15 of 17 studies reported higher odds of ectropion in the subciliary group than in the transconjunctival group [3,9,10,11,12,13,16,18,19,21,22,23,24,25,26,27,29]. However, only one study reported a marginal statistically significant result (*p* value = 0.04) [20]. The insignificant OR might be due to the wide confidence interval, which covers the OR point equal to one. This emphasizes the strength of the meta-analysis. Because we pooled all of the studies in this research, we were able to obtain a large number of cases, thus increasing the power of our statistics. Our meta-analysis revealed a significantly increased risk of postoperative ectropion with the subciliary approach compared with the transconjunctival approach. The pooled OR was 2.94 (1.63–5.31), which was similar to that reported in the meta-analysis conducted by AI-Moraissi et al., who found an OR of 3.54 (1.28–9.83) [30].

With regard to postoperative entropion, all included studies showed a lower risk of entropion in the subciliary group than in the transconjunctival group, although none of these results were statistically significant. The OR in our study was 0.17 (0.07–0.42), which is significant, as was shown in a previous meta-analysis conducted by Zhang and AI-Moraissi [2,17]. There are several reasons why the transconjunctival approach induces entropion [6,13,23,31]. First, making an incision in the conjunctiva increases the risk of conjunctival scar, shortens the posterior lamella, and inverts the eyelid. In addition, disrupting or shortening the retractors can cause inward rotation of the eyelid margin. Furthermore, anterior migration or overriding of the orbicularis muscle results in eyelid inversion.

When the lower eyelid rests too low, the white sclera is exposed, which results in scleral show. The scleral show was not significantly different between the subciliary and transconjunctival approaches in every included study. However, when we pooled the results of all these studies, scleral show was marginally significantly higher in the subciliary group. A subciliary incision often extends through or near the orbicularis oculi muscle and septum, which are critical for eyelid tone and position. In addition, healing after surgery causes fibrosis contracture in the deeper tissues, leading to eyelid retraction that exposes the inferior sclera.

Our meta-analysis also revealed a significantly higher risk of scarring in the subciliary group than in the transconjunctival group. This is similar to the findings of previous meta-analyses conducted by AI-Moraissi and Zhang [2,17]. It is noteworthy that the included studies used the subjective descriptions of surgeons or patients to define scar severity [3,10,16,18,19]. Strictly speaking, we need a standardized and objective method to evaluate scar severity. In the study conducted by Haghighat et al. in 2017, the authors applied the 10-unit visual analog scale (VAS) to quantify the appearance of the scar [25]. In Haghighat’s study, zero indicated no scar and ten was considered the worst scar possible. The use of the VAS in more research in the future would improve the comparability among studies.

When assessing the overall risk of eyelid malposition, including ectropion, entropion, scleral show, and scar, the transconjunctival approach has an advantage over the subciliary approach. Ectropion is the most common eyelid complication after the subciliary approach. Predisposing factors for postoperative ectropion in the subciliary group include old age and lower eyelid laxity. For example, in the study by Ridgway et al. conducted in 2009, only 1 of 56 patients in the subciliary group required surgery for correction of postoperative ectropion, and that patient was the oldest in the subciliary group. Ridgway et al. also suggested in that older patients with lower-lid laxity, preventative measures should be adopted to reduce the risk of ectropion, such as cheek suspension, frost/tarsorrhaphy suture, or tarsal strip procedures. In addition, conservative, postoperative management with taping and massage is generally effective in improving ectropion [16].

To the best of our knowledge, no studies have specifically examined the correlation between postoperative ectropion and visible scarring. Ectropion is commonly attributed to contracture of the anterior lamella, suggesting that scarring and ectropion may share similar underlying mechanisms. However, clinical evidence indicates that the presence of one does not necessarily imply the occurrence of the other. For example, Vaibhav and Salgarelli reported that in their subciliary groups, the rate of postoperative ectropion was zero, whereas the rates of visible scarring were 10% and 17.5%, respectively. Similarly, Hasan et al. observed that postoperative ectropion tended to resolve over time, while scarring persisted, further implying that the two complications do not always co-occur [32]. Consequently, most studies report these outcomes separately, without testing their statistical association. Future research should include both scar assessments and ectropion, followed by regression analysis to clarify their potential correlation.

It is noteworthy that reconstruction of the orbital floor likely involves greater tissue exposure and tension, which may increase the risk of eyelid complications compared with surgeries limited to the infraorbital rim. Consequently, inclusion of patients with or without orbital floor reconstruction could confound complication rates. In our analysis, however, the majority of included studies (15 of 19) involved orbital floor reconstruction. Importantly, exclusion of the four studies without orbital floor reconstruction did not alter the overall results.

The limitation of our research is that we included only six randomized clinical studies. Most of the published studies had a retrospective design and might have confounding variables in the decision as to which surgical approach to perform. Prospective, randomized clinical studies are needed in the future; therefore, meta-analysis including more randomized studies could result in more objective conclusions. The strengths of our research are its completeness, as we pooled a large number of cases to provide convincing evidence. Our results can serve to remind surgeons to be aware of the risks of various eyelid complications before surgery and communicate meticulously with patients during the decision-making process.

## 5. Conclusions

This meta-analysis demonstrated that the subciliary approach for orbital and zygomaticofacial fracture repair carries significantly higher risks of postoperative ectropion, scleral show, and visible scarring, whereas the transconjunctival approach is associated with a greater risk of entropion. These findings underscore that no single approach is without complications, and surgical decisions should balance the advantages of exposure with the potential for eyelid malposition. Importantly, most of the included studies involved orbital floor reconstruction, and excluding those without such procedures did not alter the results, suggesting robustness of the findings. Nevertheless, the predominance of retrospective studies highlights the need for additional prospective, randomized trials using standardized outcome measures, particularly for scar assessment. Until such evidence is available, our results can serve to guide surgeons in selecting the most appropriate surgical approach and in counseling patients regarding expected postoperative outcomes.

## Figures and Tables

**Table 1:** Risk of bias and quality assessment of included studies.

Study	Selection				Comparability		Outcomes			
	Representativeness of Cohort	Selection of Nonexposed Cohort	Ascertainment of Exposure	Outcome Lacking at the Beginning	Main Factor	Additional Factor	Outcome Assessment	Sufficient Follow-Up Time	Follow Up Adequacy	Score ^a^
Bronstein [9]	★	★	★	★	★	☆	★	★	★	8
Mohamed [10]	★	★	★	★	★	★	★	★	★	9
Appling [11]	★	★	★	★	★	☆	★	★	★	8
Patel [3]	★	★	★	★	★	☆	★	★	★	8
Mehrnoush [12]	★	★	★	★	★	★	★	★	★	9
Ridgeway [16]	★	★	★	★	★	☆	★	★	★	8
Trevisiol [13]	★	★	★	★	★	☆	★	★	★	8
Neovius [22]	★	★	★	★	★	☆	★	★	★	8
Giraddi [26]	★	★	★	★	★	★	★	★	★	9
Haghighat [25]	★	★	★	★	★	☆	★	★	★	8
Vaibhav [18]	★	★	★	★	★	★	★	★	★	9
Pausch [21]	★	★	★	★	★	☆	★	★	★	8
Kesselring [23]	★	★	★	★	★	☆	★	★	★	8
Ishida [24]	★	★	★	★	★	☆	★	★	★	8
Salgarelli [19]	★	★	★	★	★	☆	★	★	★	8
Raschke [20]	★	★	★	★	★	☆	★	★	★	8
Bhatti [27]	★	★	★	★	★	☆	★	★	★	8
Subramanian [28]	★	★	★	★	★	★	★	★	★	9
EI-Anwar [29]	★	★	★	★	★	★	★	★	★	9

^a^ The total score of Newcastle-Ottawa scale is 9 points; ★ represents that the item has received a score, while ☆ represents that it has not.

**Table 2:** Demographic characteristics of patients in studies included in meta-analysis.

Authors	Year	Study Design	Age	Groups	Number of Pts	Follow-Up
Bronstein et al. [9]	2020	Retrospective	34.8 ± 12.4	Subciliary	82	at least 6 months
			35.3 ± 11.9	Transconjunctival	102	
Mohamed et al. [10]	2020	Randomized clinical study	30.9 ± 12.6	Subciliary	15	6 months
			37.4 ± 9.0	Transconjunctival	15	
Appling et al. [11]	1993	Retrospective	11–60	Subciliary	25	6 weeks–5 years
				Transconjunctival	33	
Patel et al. [3]	1998	Retrospective	12–63	Subciliary	30	at least 8 months
				Transconjunctival		
Mehrnoush et al. [12]	2021	Randomized clinical study	34.6 ± 14.2	Subciliary	42	5 months
			29.0 ± 9.0	Transconjunctival	38	
Ridgeway et al. [16]	2009	Retrospective	39	Subciliary	56	6 weeks
				Transconjunctival	45	
Trevisiol et al. [13]	2021	Retrospective	44	Subciliary	36	12–74 months
				Transconjunctival	33	
Neovius et al. [22]	2017	Retrospective	41	Subciliary	37	at least 6 months
				Transconjunctival	91	
Giraddi et al. [26]	2012	Randomized clinical study	28.4	Subciliary	10	3 months
				Transconjunctival	10	
Haghighat et al. [25]	2010	Prospective clinical study	26.7 ± 6.5	Subciliary	17	4 weeks
				Transconjunctival	17	
Vaibhav et al. [18]	2016	Randomized clinical study	20–60	Subciliary	20	3 months
				Transconjunctival	20	
Pausch et al. [21]	2016	Retrospective	42.7 ± 21.1	Subciliary	225	6 months
				Transconjunctival	121	
Kesselring et al. [23]	2016	Retrospective	37.5	Subciliary	47	NR
				Transconjunctival	26	
Ishida et al. [24]	2016	Retrospective	NR	Subciliary	29	6 weeks–6.8 years
				Transconjunctival	179	
Salgarelli et al. [19]	2010	Retrospective	37.1	Subciliary	219	6–48 months
				Transconjunctival	32	
Raschke et al. [20]	2012	Retrospective	43.3 ± 19.0	Subciliary	114	9 months
				Transconjunctival	197	
Bhatti et al. [27]	2022	Prospective clinical study	32.8	Subciliary	28	5 months
				Transconjunctival	22	
Subramanian [28]	2009	Randomized clinical study	NR	Subciliary	10	at least 6 months
				Transconjunctival	10	
EI-Anwar et al. [29]	2017	Randomized clinical study	31.3 ± 9.2	Subciliary	20	6 weeks
			31.6 ± 7.7	Transconjunctival	20	

Pts, patients; NR, not reported; age is presented as mean ± SD, mean, or range.

**Table 3:** Complications in studies included in meta-analysis.

Study	Groups	Num of pts	Orbital Floor Reconstruction	Types of Complication (%)
Bronstein, 2020 [9]	Sub ciliary	82	Yes	ectropion (2.4), entropion (1.2), lagophthalmos (1.2), corneal injury (7.3), keratoconjunctivitis sicca (3.7)
	Transconjunctival	102		ectropion (2.0), entropion (3.9), lagophthalmos (1.0), corneal injury (6.9), keratoconjunctivitis sicca (3.9)
Mohamed, 2020 [10]	Subciliary	15	Yes	ectropion (20), entropion (0), scleral show (26.7), visible scar (13.3) ^a^
	Transconjunctival	15		ectropion (6.7), entropion (20), scleral show (13.3), visible scar (0) ^a^
Appling, 1993 [11]	Subciliary	25	Yes	ectropion (12.0), scleral show (28.0), canthal malposition (0)
	Transconjunctival	33		ectropion (0), scleral show (3.0), canthal malposition (9.1)
Patel, 1998 [3]	Subciliary	30	Yes	ectropion (6.7), entropion (0), scleral show (20), visible scar (6.7) ^b^
	Transconjunctival	30		ectropion (0), entropion (0), scleral show (3.3), visible scar (0) ^b^
Mehrnoush, 2021 [12]	Subciliary	42	Yes	ectropion (2.4), entropion (0), epiphora (7.1), scleral show (11.9)
	Transconjunctival	38		ectropion (13.2), entropion (5.3), epiphora (23.7), scleral show (18.4)
Ridgeway, 2009 [16]	Subciliary	56	Yes	Ectropion (12.5), entropion (0), scar (3.6) ^c^, lid edema (8.9)
	Transconjunctival	45		Ectropion (0), entropion (4.4), scar (0) ^c^, lid edema (0)
Trevisiol, 2021 [13]	Subciliary	36	Yes	ectropion (8.3), entropion (0)
	Transconjunctival	33		ectropion (0), entropion (0)
Neovius, 2017 [22]	Subciliary	37	Yes	ectropion (8.1), entropion (0), scleral show (11.0), canthal malposition (0)
	Transconjunctival	91		ectropion (2.2), entropion (0), scleral show (4.4), canthal malposition (2.2)
Giraddi, 2012 [26]	Subciliary	10	Yes	ectropion (30.0), entropion (0), laceration of tarsal plate (0), buttonhole laceration of lower eyelid (10.0)
	Transconjunctival	10		ectropion (10.0), entropion (30.0), laceration of tarsal plate (10.0), buttonhole laceration of lower eyelid (0)
Haghighat, 2017 [25]	Subciliary	17	Yes	ectropion (17.6), scar (3.7 ± 0.6) ^d^
	Transconjunctival	17		ectropion (0), scar (0.0 ± 0.0) ^d^
Vaibhav, 2016 [18]	Subciliary	20	No	ectropion (0), entropion (0), unsatisfactory scar (10) ^e^
	Transconjunctival	20		ectropion (0), entropion (5), unsatisfactory scar (0) ^e^
Pausch, 2016 [21]	Subciliary	225	Yes	ectropion (3.6), entropion (0), eyelid retraction (0)
	Transconjunctival	121		ectropion (0), entropion (2.5), eyelid retraction (0)
Kesselring, 2016 [23]	Subciliary	47	Yes	ectropion (2.1), entropion (0)
	Transconjunctival	26		ectropion (0), entropion (0)
Ishida, 2016 [24]	Subciliary	29	Yes	ectropion (6.9), scleral show (6.9)
	Transconjunctival	179		ectropion (0.6), entropion (3.4), symblepharon (1.7), trichiasis (1.1), lacrimal canaliculus avulsion (1.1), conjunctival granulation (2.2), canthal malposition (0.6)
Salgarelli, 2010 [19]	Subciliary	219	Yes	ectropion (0), visible scar (17.5) ^f^, scleral show (1.3)
	Transconjunctival	32		ectropion (0), visible scar (3) ^f^, scleral show (0)
Raschke, 2012 [20]	Subciliary	114	Yes	ectropion (5.3), entropion (0), scleral show (21.8)
	Transconjunctival	197		ectropion (1.0), entropion (1.0), scleral show (6.6)
Bhatti, 2022 [27]	Subciliary	28	No	ectropion (14.2), entropion (0), scleral show (11.0), epiphora (21.0)
	Transconjunctival	22		ectropion (13.5), entropion (9.1), scleral show (17.0), epiphora (20.0)
Subramanian, 2009 [28]	Subciliary	10	No	ectropion (0), scar (1.55) ^g^
	Transconjunctival	10		ectropion (10), scar (1.00) ^g^, prolonged edema (20)
EI-Anwar, 2017 [29]	Subciliary	20	No	ectropion (10), entropion (0), scleral show (15), intolerable pain (10)
	Transconjunctival	20		ectropion (0), entropion (20), scleral show (0), intolerable pain (15)

pts patients; ^a^ visible scar scale (5-point ordinal scale) created by Feldman; ^b^ visible scar scale (binary). ^c^ hypertrophic (or visible) scar scale (binary); ^d^ VAS score for scar; ^e^ patient-reported satisfaction scale (binary); ^f^ visible scar scale (binary); ^g^ visible scar scale (3-point grading) created by the authors—1 point for invisible scar, 2 for barely visible scar, and 3 for visible scar.

**Table 4:** Summary of Scar Evaluation Methods in Studies.

Study	Scar Assessment Method	Score Range
Mohamed, 2020 [10]	Feldman scar esthetic score	0–4 (0 = Scar not visible, 1 = Barely visible, 2 = Noticeable, 3 = Very noticeable, 4 = Extremely noticeable
Patel, 1998 [3]	visible scar scale (binary)	yes/no
Ridgway, 2009 [16]	hypertrophic (or visible) scar scale	yes/no
Vaibhav, 2016 [18]	patient-reported satisfaction scale (binary)	satisfactory/unsatisfactory
Salgarelli, 2010 [19]	visible scar scale (binary)	yes/no
Subramanian, 2009 [28]	VAS (3-point visibility score)	custom; 1 = invisible, 2 = barely visible, 3 = visible

Vas, visual analog scale.

## Data Availability

Data analyzed in this study were a re-analysis of existing data openly available in works cited in the reference section.

## Supplementary Materials

The following supporting information can be downloaded at: https://www.mdpi.com/article/10.3390/jcm14186431/s1, Table S1: PRISMA 2020 Checklist; Table S2: Excluded studies and reasons.

## Abbreviations

The following abbreviations are used in this manuscript:

Ors	Odds Ratios
Cis	Confidence Intervals
VAS	Visual Analog Scale
