# Supplementary material for: Eyelid Complications in Subciliary Versus Transconjunctival Approaches to Orbital and Zygomaticofacial Fractures: A Meta-Analysis
Source: J Clin Med. 2025 Sep 12;14(18):6431. doi: 10.3390/jcm14186431 (PMC12470683; doi:10.3390/jcm14186431)
Supplement: Supplementary file 1 [file jcm-14-06431-s001.zip › Supplementary Table S2_exclusion reasons.pdf]

Table S2 - Excluded studies and reasons

Citations	Reasons
Palavalli MH, Huayllani MT, Gokun Y, Lu Y, Janis JE. Surgical Approaches to Orbital Fractures: A Practical and Systematic Review. *Plast Reconstr Surg Glob Open* 2023; 11(5): e4967.	A systematic review.
Al-Moraissi E, Elsharkawy A, Al-Tairi N, Farhan A, Abotaleb B, Alsharaee Y et al. What surgical approach has the lowest risk of the lower lid complications in the treatment of orbital floor and periorbital fractures? A frequentist network meta-analysis. J Craniomaxillofac Surg 2018; 46(12): 2164-2175.	A review and network meta-analysis.
Kothari NA, Avashia YJ, Lemelman BT, Mir HS, Thaller SR. Incisions for orbital floor exploration. *J Craniofac Surg* 2012; 23(7 Suppl 1): 1985-1989.	A systematic review.
Zhang J, He X, Qi Y, Zhou P. The better surgical timing and approach for orbital fracture: a systematic review and meta-analysis. *Ann Transl Med* 2022; 10(10): 564.	A systematic review and meta-analysis.
Al-Moraissi EA, Thaller SR, Ellis E. Subciliary vs. transconjunctival approach for the management of orbital floor and periorbital fractures: A systematic review and meta-analysis. *J Craniomaxillofac Surg* 2017; 45(10): 1647-1654.	A systematic review and meta-analysis.
Bergler W, Hoffmann A, Hörmann K. [Late sequelae of lateral and central mid-facial fractures after osteosynthesis with miniplates]. *Hno* 1997; 45(3): 128-132.	Not written in English; focus on the sequelae of miniplates.
Bertram G, Luckhaupt H, Rose KG. [The expanded transconjunctival approach with lateral canthotomy to the orbital margin, floor and infraorbital nerve]. *Hno* 1991; 39(4): 134-137.	Not written in English; focus on the Transconjunctival approach.
Stoll W, Busse H, Kroll P. [Transconjunctival incision and lateral canthotomy. A suitable approach for orbital floor and zygomatic bone correction]. *Laryngol Rhinol Otol (Stuttg)* 1984; 63(2): 45-47.	Not written in English; it only had the transconjunctival incision group, without comparing to the subciliary incision group.
Harish KM, Bhagat JA, Tulasidas G. A Case Report of Scarless Direct Access to the Infraorbital Rim Using a Retroseptal Transconjuctival Approach. *Cureus* 2019; 11(1): e3836.	A case report; it only had one case.
